# Children and Adults with Refractory Acute Graft-versus-Host Disease Respond to Treatment with the Mesenchymal Stromal Cell Preparation “MSC-FFM”—Outcome Report of 92 Patients

**DOI:** 10.3390/cells8121577

**Published:** 2019-12-05

**Authors:** Halvard Bonig, Zyrafete Kuçi, Selim Kuçi, Shahrzad Bakhtiar, Oliver Basu, Gesine Bug, Mike Dennis, Johann Greil, Aniko Barta, Krisztián M. Kállay, Peter Lang, Giovanna Lucchini, Raj Pol, Ansgar Schulz, Karl-Walter Sykora, Irene Teichert von Luettichau, Grit Herter-Sprie, Mohammad Ashab Uddin, Phil Jenkin, Abdulrahman Alsultan, Jochen Buechner, Jerry Stein, Agnes Kelemen, Andrea Jarisch, Jan Soerensen, Emilia Salzmann-Manrique, Martin Hutter, Richard Schäfer, Erhard Seifried, Shankara Paneesha, Igor Novitzky-Basso, Aharon Gefen, Neta Nevo, Gernot Beutel, Paul-Gerhardt Schlegel, Thomas Klingebiel, Peter Bader

**Affiliations:** 1 Goethe University Medical Center, Institute of Transfusion Medicine and Immunohematology, and German Red Cross Blood Center Frankfurt, Frankfurt am Main, Frankfurt 60528, Germany; r.schaefer@blutspende.de (R.S.); e.seifried@blutspende.de (E.S.); 2 Division for Stem Cell Transplantation and Immunology, Department for Children and Adolescents, University Hospital Frankfurt, Frankfurt am Main, Frankfurt 60590, Germany; zyrafete.kuci@kgu.de (Z.K.); selim.kuci@kgu.de (S.K.); shahrzad.bakhtiar@kgu.de (S.B.); andrea.jarisch@kgu.de (A.J.); jan.soerensen@kgu.de (J.S.); emilia.salzmann@kgu.de (E.S.-M.); martin.hutter@kgu.de (M.H.); thomas.klingebiel@kgu.de (T.K.); peter.bader@kgu.de (P.B.); 3 University Children’s Hospital Essen, Essen 45122, Germany; o.Basu@uk-essen.de; 4 Department of Medicine 2, Hematology and Oncology, University Hospital, Goethe University Frankfurt, Frankfurt am Main 60590, Germany; gesine.bug@kgu.de; 5 Christie Hospital, Department of Haematology, Manchester M20 4BX, UK; Mike.Dennis@christie.nhs.uk; 6 University Children’s Hospital Heidelberg, Heidelberg 69120, Germany; Johann.Greil@med.uni-heidelberg.de; 7 Central Hospital of Southern Pest, National Institute of Hematology and Infectious Diseases, Department for Haematology and SCT, Budapest H1097, Hungary; aniko.barta@gmail.com; 8 Central Hospital of Southern Pest, National Institute of Hematology and Infectious Diseases, Pediatric Hematology and Stem Cell Transplantation Department, Budapest H1097, Hungary; dr.kallay@gmail.com; 9 University Children’s Hospital Tübingen, Tübingen 72076, Germany; peter.lang@med.uni-tuebingen.de; 10 Great Ormond Street Hospital, Department of Hematology/Oncology, London WC1N 3JH, UK; Giovanna.Lucchini@gosh.nhs.uk; 11 Department of Haematology, University of Sheffield, Sheffield S10 2TN, UK; rajpol8@gmail.com; 12 Department of Pediatrics, University Medical Center Ulm, Ulm 89070, Germany; ansgar.schulz@uniklinik-ulm.de; 13 Children’s Hospital, Medizinische Hochschule Hannover, Hannover 30625, Germany; Sykora.Karl-Walter@mh-hannover.de; 14 Division of Pediatric Hematology/Oncology, Department of Pediatrics, Kinderklinik München Schwabing, Klinikum Rechts der Isar, Technische Universität München, München 80804, Germany; Irene.Teichert-vonLuettichau@mri.tum.de; 15 Department I of Internal Medicine, Center for Integrated Oncology Aachen Bonn Cologne Duesseldorf, Center for Molecular Medicine Cologne, University of Cologne, Cologne 50937, Germany; grit.herter-sprie@uk-koeln.de; 16 Department for Stem Cells & Immunotherapies, NHSBT, Birmingham B15 2SG, UK; Ash.Uddin@nhsbt.nhs.uk (M.A.U.); phil.jenkin@nhsbt.nhs.uk (P.J.); 17 Department of Pediatric Hematology/Oncology, King Abdullah Specialist Children’s Hospital, Riyadh 14611, Saudi Arabia; aalsultan@gmail.com; 18 Oslo University Hospital, Department of Pediatric Hematology and Oncology, Oslo 0424, Norway; jocbuc@ous-hf.no; 19 Schneider Children’s Medical Center of Israel, Department for Hemato-Oncology, Petach Tikva 4920235, Israel; jstein@clalit.org.il; 20 B-A-Z County Hospital, Pediatric Haematology and Stem Cell Transplantation Unit, Miskolc 3526, Hungary; kelemen.igyek@bazmkorhaz.hu; 21 Department of Haematology & Stem Cell Transplantation, Birmingham Heartlands Hospital, Birmingham B9 5SS, UK; shankara.paneesha@nhs.net; 22 Queen Elizabeth University Hospital, Glasgow, Glasgow G51 4TF, UK; novitzky@outlook.com; 23 Rambam Medical Center, Ruth Rappaport Children’s Hospital, Pediatric Hematology Oncology Division, The Reiner-Shudi Pediatric Bone Marrow Transplantation Unit, Haifa 3109601, Israel; r_gefen@rambam.health.gov.il (A.G.); n_nevo@rambam.health.gov.il (N.N.); 24 Hannover Medical School (MHH), Hannover, Department of Hematology, Hemostasis, Oncology and Stem Cell Transplantation, Hannover 30625, Germany; Beutel.Gernot@mh-hannover.de; 25 University Children’s Hospital Würzburg, Würzburg 97080, Germany; Schlegel_P@ukw.de

**Keywords:** graft-versus host, transplantation, mesenchymal stromal cell, cell therapy, hospital exemption, steroid-resistant aGvHD, refractory aGvHD

## Abstract

(1) Background: Refractory acute graft-versus-host disease (R-aGvHD) remains a leading cause of death after allogeneic stem cell transplantation. Survival rates of 15% after four years are currently achieved; deaths are only in part due to aGvHD itself, but mostly due to adverse effects of R-aGvHD treatment with immunosuppressive agents as these predispose patients to opportunistic infections and loss of graft-versus-leukemia surveillance resulting in relapse. Mesenchymal stromal cells (MSC) from different tissues and those generated by various protocols have been proposed as a remedy for R-aGvHD but the enthusiasm raised by initial reports has not been ubiquitously reproduced. (2) Methods: We previously reported on a unique MSC product, which was generated from pooled bone marrow mononuclear cells of multiple third-party donors. The products showed dose-to-dose equipotency and greater immunosuppressive capacity than individually expanded MSCs from the same donors. This product, MSC-FFM, has entered clinical routine in Germany where it is licensed with a national hospital exemption authorization. We previously reported satisfying initial clinical outcomes, which we are now updating. The data were collected in our post-approval pharmacovigilance program, i.e., this is not a clinical study and the data is high-level and non-monitored. (3) Results: Follow-up for 92 recipients of MSC-FFM was reported, 88 with GvHD ≥°III, one-third only steroid-refractory and two-thirds therapy resistant (refractory to steroids plus ≥2 additional lines of treatment). A median of three doses of MSC-FFM was administered without apparent toxicity. Overall response rates were 82% and 81% at the first and last evaluation, respectively. At six months, the estimated overall survival was 64%, while the cumulative incidence of death from underlying disease was 3%. (4) Conclusions: MSC-FFM promises to be a safe and efficient treatment for severe R-aGvHD.

## 1. Introduction

Much progress has been made in allogeneic transplantation. Improvements include more refined HLA typing, larger donor registries providing better-matched donors, better insight into graft-versus-host disease (GvHD)-associated donor properties and better options for GvHD prophylaxis. Yet the number of patients developing severe refractory acute GvHD (R-aGvHD) has not decreased. Approximately one in nine transplant recipients will develop this complication. Thus, approximately 1800 patients/year in Europe and 4000 patients/year world-wide, will be affected. For these patients, prognosis remains guarded as a wide panel of immunosuppressive medicines have been explored and failed [1]. The most recent large study estimates the 6-month and 4-year survival of R-aGvHD at 40% and 15%, respectively [2], in agreement with a host of other published work.

Seminal work by the Swedish group in 2003 firmly established the conviction that mesenchymal stromal cells (MSCs) could provide control over R-aGvHD [3]. Initial data seemed to confirm this notion [4,5,6,7,8,9,10]. However, this impression was only partly supported by recent, more systematic clinical experience: The only commercial MSC trial failed to show lasting clinical benefit, let alone improved survival [11]. Several, more recent academically-led studies or case series have also failed to convincingly demonstrate meaningful survival benefits [12,13,14,15,16]. However, ample data has established that not all MSC products are created equal. Particularly, donor-to-donor variability [17], number of ex-vivo population doublings [18,19] and methods of MSC manufacturing significantly affecting in vivo and in vitro properties [20]. The product used here, licensed in Germany with National Marketing Authorization (hospital exemption), for which post-approval safety/efficacy data were systematically collected and which are reported here, differs from other MSC products in several salient ways, which in aggregate are believed to qualify its uniqueness. The product is generated not from bone marrow (BM) of single donors one at a time, but by pooling mononuclear bone marrow cells (BM-MNCs) from eight randomly selected donors. Presence of BM-MNC from multiple incompatible donors in cell culture induces a strong-multi-directional allogeneic reaction. This presumably either selects particularly potent MSCs or poises them towards a more strongly immunomodulatory phenotype than selection and expansion of single-donor MSCs. Thus, we previously reported that the ensuing MSC product possessed significantly greater suppressive activity in a mixed lymphocyte reaction than the mean of MSCs individually raised from the same eight donors, or a pool of MSCs from these eight individual MSC donor products [21]. Moreover, expansion of all clinical batches from the same MSC bank guarantees equal potency of all MSC end products [21]. Importantly, this “pharmaceutical” approach guarantees that no potentially MSC-responsive patient is inadvertently treated with poorly efficacious MSCs. The MSC product is mitotically young, end of passage 2 [21], and raised in platelet lysate, i.e., completely xeno-free; both variables are apparently associated with favorable pharmacological efficacy [7,22,23,24,25,26].

Ninety-two allo-transplant recipients of either sex and of any age, donor type, transplant source, conditioning or immunosuppression, with severe, mostly ≥grade III, R-aGvHD received a median (range) of three (1–9) doses of MSC-FFM (MSC-Frankfurt on Main; the proprietary name of the specific medicinal product, is used here throughout to distinguish statements about this particular product from statements about MSCs in general); clinical follow-up was requested as part of our legally mandated pharmacovigilance activity. Response and survival data are reported.

## 2. Materials and Methods

### 2.1. Mesenchymal Stromal Cells-Frankfurt on Main (MSC-FFM)

Development of the proprietary manufacturing process of MSC-FFM was previously reported in detail [21,27]. In brief, MSC-FFM is a cryopreserved dispersion of BM-derived MSC at a concentration of 1–3 × 10^6^ mL in normal saline supplemented with 5% *w*/*v* human albumin and 10% *v*/*v* DMSO. The MSCs are generated from pooled, previously cryopreserved mononuclear cells from eight random donor bone marrow (BM) aspirates by plastic adherence, expanded to near-confluence and cryopreserved in small aliquots. The clinical product was manufactured by expanding individual aliquots through two additional passages and subsequent freezing in clinically useful doses. Quality attributes, both specification-defining and ancillary, were previously reported [27]. MSC-FFM is manufactured according to GMP and all applicable laws and regulations. It is released by a qualified Person with permission from the German biological medicines agency Paul-Ehrlich-Institute for use within the scope of a hospital exemption on the German market. 

MSC-FFM is recommended for use at 1–2 × 10^6^ kg, i.v., in four weekly applications as rapid infusion immediately after thawing. MSC-FFM has no known contraindications or cross-reactivities, although we recommend avoidance of inhibitors of prostaglandin synthesis due to the partial dependence of anti-inflammatory effects of MSC-FFM on PGE2 [21]. During the course of MSC-FFM other GvHD modifying agents may be continued; steroids were tapered as clinically permitted.

MSC-FFM is labeled for R-aGvHD after allogeneic transplantation of any kind and for any underlying disease, without age restriction. Children and adults were eligible for MSC-FFM as soon as aGvHD was clinically manifest (see below for aGvHD grading), irrespective of leading target organ system and had demonstrated steroid refractoriness. Eligibility was not restricted by the presence of any other complications, graft type, quality of donor-recipient MHC match, conditioning, underlying disease and co-medication for aGvHD. Thereafter, patients remained eligible irrespective of the number, type and aggregate durations of further lines of immunosuppressants that were used after establishment of steroid refractoriness. As despite its National Marketing Authorization MSC-FFM was not formally marketed due to reimbursement issues, transplant centers ordered MSC-FFM directly from the manufacturer. At that time, some minimal patient and transplant characteristics as well as staging and prior treatment of aGvHD had to be submitted. The label does not limit MSC-FFM to severe R-aGvHD. However, with four exceptions all suffered from aGvHD °III or higher (1 patient: not specified), presumably largely because of reimbursement.

### 2.2. Data Collection and Analysis

Data on adverse events and clinical outcomes were collected as part of the Pharmaceutical Manufacturer’s legally mandated continuous pharmacovigilance effort. Short structured questionnaires were sent to transplant centers, but participation was not enforced. Data collected included patient demographics, graft type and conditioning, onset, severity and treatment of aGvHD before, during and after MSC-FFM treatment, adverse events during and after MSC-FFM infusion, as well as relapse and death including cause, as applicable. Responses were not monitored. The first evaluation, intended to document the day-28-response, which was previously shown to predict survival, was provided after a median of 31 days (interquartile range, 28–47 days; range 12–370 days). Data were entered into a clinical outcomes database and queried for clinical response rates and survival for the entire cohort as well as for sub-cohorts as defined above. Available information was high-level and limited to that provided in this manuscript.

### 2.3. Definitions, Stratifications and Statistical Analysis

aGvHD diagnosis and severity scoring: Primarily, aGvHD was diagnosed clinically; histological or other non-clinical evidence was only sought to rule out alternative diagnoses in unclear cases. Acute GvHD scoring followed the Seattle-Glucksberg modified criteria [23,28]. 

Patients were stratified by age (age ≤/>18 years), by aGvHD severity as well as by number of failed treatments in addition to cortisone. The standard definition of steroid refractoriness is aGvHD which has worsened after 3 days or failed to improve after 5 days of systemic high-dose steroids [29]. In this manuscript, the R-aGvHD patients were further sub-categorized as SR-aGvHD (SR=“steroid-refractory”; unresponsiveness to cortisone and up to one additional, concomitantly used immunosuppressant) or treatment-refractory (TR-) aGvHD (failing steroids plus ≥2 additional lines of immunosuppressive medicines). 

Responses were categorized as complete (CR), i.e., complete resolution of all signs of aGvHD (grade 0), partial (PR) in patients who showed aGvHD reduction by at least one full grade according to the Glucksberg criteria, or none (NR). The sum of CR and PR was referred to as overall response (OR). Response and survival were assessed at two time points. Half of the patients had their first evaluation before day 31 with an interquartile range of 28–47 days. The second evaluation was performed at last follow-up. 

Median survival follow-up time since first MSC infusion was calculated using the reverse Kaplan–Meier method. OS probability was estimated using Kaplan–Meier statistics. Survival time was considered from the date of the first MSC infusion to date of death or last follow-up (LFU; for censored patients). Cumulative incidence curves were used to estimate death from underlying disease considering death from other causes as a competing risk. Results were given as a probability or cumulative incidences with 95% confidence intervals. Six-month predicted estimates for OS and cumulative incidences were considered as described. Univariate Cox proportional-hazard regression model was performed to investigate if time of onset of aGVHD, time between onset of aGVHD until date of first MSC infusion and number of therapy lines prior to MSC were associated with reaching CR at first evaluation. The endpoint was CR. The time was considered from first MSC infusion to date of first evaluation.

All tests were two-tailed; a p-value of <0.05 was considered statistically significant. Statistical analyses were performed using the statistical software R, version 3.5.3 (R Project for statistical computing, www.r-project.org/) [30]. 

## 3. Results

### 3.1. Patients

This manuscript reports outcome data from all R-aGvHD patients treated with MSC-FFM up to the transfer of the product line to a commercial pharmaceutical manufacturer in 2018, i.e., including the previously reported ones [27]. Data cut-off was 1 May 2018, at which time the median follow-up was 11.0 months (range: 0.5–77.8). Ninety-two (92) patients with R-aGvHD were from 23 allogeneic transplant units based in six countries (Germany, Hungary, Israel, Norway, Saudi-Arabia and UK) received MSC-FFM.

**Figure 1:**
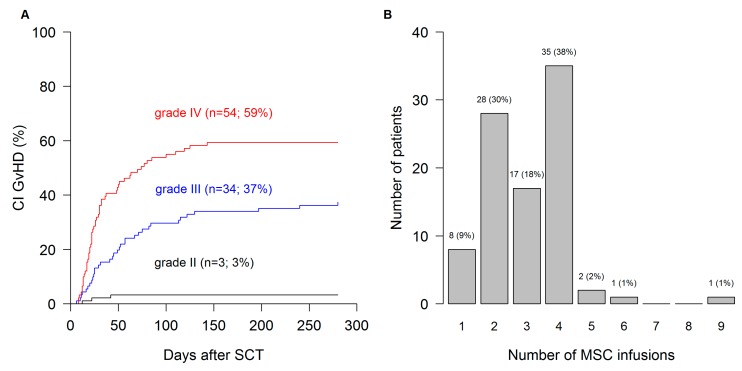
Onset of aGvHD and number of MSC infusions. (**A**) Median overall aGvHD onset was 30 days (range 6–280 days). (**B**) Median number of doses of MSC-FFM was 3 (range: 1–9), with the majority receiving either 2 or, as intended per label, 4 doses.

Limited baseline and follow-up data were provided for all patients. Two thirds were children/adolescents, three quarters had a malignant disease as an indication for transplantation. Half of the patients each received BM and PBSC, 84% from matched donors. One quarter of the patients each underwent conditioning with TBI, Treosulfan, Busulfan or Fludarabine. Two thirds received serotherapy, most patients developed GvHD despite immunosuppressive prophylaxis, predominantly with CSA-based regimens. Of all patients receiving MSC-FFM, 96% had severe (≥grade III) aGvHD; before the MSC-FFM infusion, the majority had several additional lines of immunosuppressants, as many as 12, besides steroids. Detailed data are shown in Table 1. Median aGvHD onset was 30 days with a very wide range (6–280 days), similarly for grade III and grade IV GvHD (Figure 1A). Between onset of aGvHD and the first MSC-FFM infusion, a median of 28 days elapsed (range, 4–380 days).

### 3.2. MSC-FFM Treatment and Tolerability

MSC-FFM was delivered cryopreserved for rapid intravenous infusion immediately after thawing in a suitable cell thawing device. A median of 3 (range: 1–9) doses of 0.6–4.5 million/kg MSCs was administered at approximately one-week intervals (Figure 1B for details). No acute infusional toxicity was reported.

### 3.3. aGvHD Outcomes

**Figure 2:**
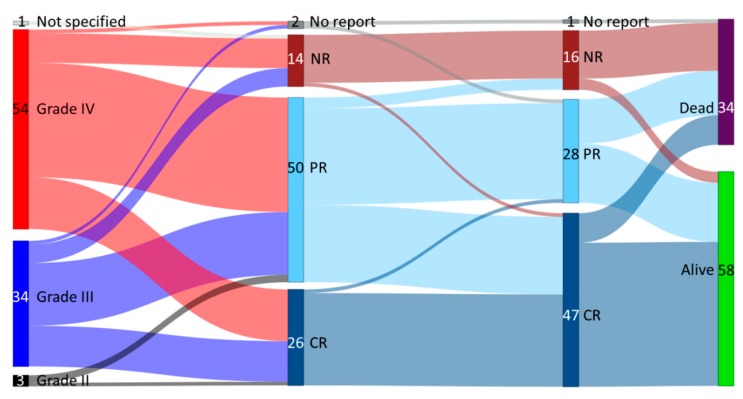
Sankey diagram of overall response and survival by severity over time. aGvHD grades II, III and IV are shaded black, ultramarine and red, respectively; the width of each bar represents their relative frequency within the cohort. Quality of response at first follow-up (2nd column from left) and at last follow-up (LFU, 3rd column from left) is depicted in Prussian blue (CR), baby blue (PR), maroon (NR) or grey (no report). In the right-most bar, survivors are shown in green, deaths in purple. The connectors’ relative width depicts the relative frequency of a specific outcome for the cohort from which the connector originates. Thus, almost half of the grade III (ultramarine to Prussian blue vs. ultramarine to baby blue) but less than one third of the grade IV (red to Prussian blue vs. red to baby blue) had achieved CR at first follow-up. The probability of a non-response was similar for grades III and IV. Between first and long-term follow-up, most CR patients remained in CR and many partial responders improved to CR (baby blue to Prussian blue), while there were very few recurrences of aGvHD in patients with clinical responses at first follow-up. The prognosis quoad vitam of non-responders at LFU was guarded (maroon to green vs. maroon to purple).

**Figure 3:**
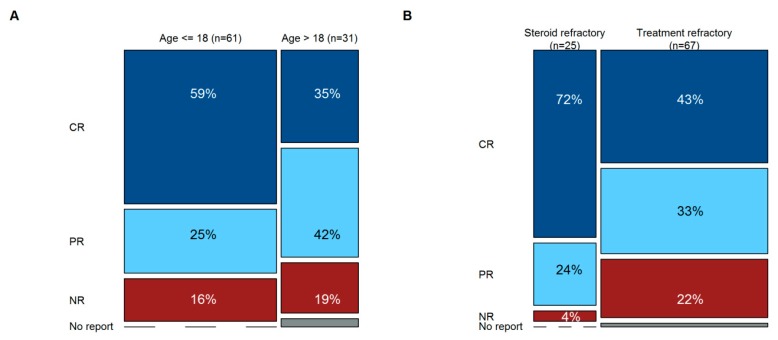
Response (LFU) by age and degree of refractoriness. The Y-axis shows the quality of response (top to bottom: Prussian blue, CR; baby blue, PR; maroon, NR; grey, no report); the height of each box represents the frequency for this cohort. The X-axis shows children/adolescents vs. adults (**A**) or only SR-aGvHD vs. TR-aGvHD (**B**). The width of the columns represents the distribution between these groups. (**A**) Response rates in pediatric vs. adult patients were similar, although CR seemed to be more frequently observed in the pediatric group. (**B**) Almost all SR-aGvHD patients responded to MSC-FFM, whereas 22% of TR-aGvHD patients were non-responders. None of the potential differences between groups were statistically significant.

**Figure 4:**
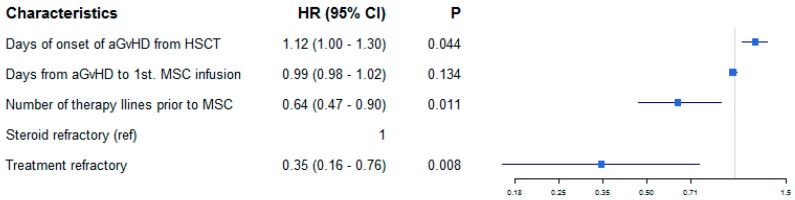
Greater number of pre-treatments is a risk factor for an inferior clinical response. Forest plot showing the strongly significant association between number of therapies before MSC-FFM and complete response (Hazard ratio 0.64; 95% CI 0.47–0.90; *p* = 0.011) and time from transplantation to onset of aGvHD and CR. Patients with a higher number of prior treatments were less likely to reach a complete response. However, association between time from aGvHD onset to first MSC infusion and complete response was not significant.

The overall response rate to MSC-FFM was 82% and 81% at first and last follow-up, respectively. Relapse of aGvHD was infrequent. First follow-up overall response was highly predictive of long-term response in that there were very few late responders thereafter, but the quality of the response improved over time, as there were 28% complete responses after first evaluation but 51% at last follow-up (see Sankey diagram in Figure 2). The probability and quality of response were largely independent of aGvHD severity and similarly good in children/adolescents vs. adults (Figure 3A). A 96% overall response and 72% complete response at last follow-up was achieved in SR-only patients receiving MSC-FFM. Patients with TR-aGvHD (two-thirds of the patients) also responded to MSC-FFM, with a 76% overall response probability including 43% complete responses at last follow-up (Figure 3B). However, the odds of achieving a complete remission in the latter compared to the “only” SR-aGvHD patients were significantly unfavorable, with a hazard ratio of 0.35 (95% CI 0.16–0.76, *p* = 0.008) (Figure 4). In other words, patients with a higher number of prior treatments were less likely to reach CR. Onset of aGvHD was also significant associated with CR (HR = 1.12, 95% CI 1.0–1.3, *p* = 0.044). By contrast, time from aGvHD onset to first MSC infusion was not associated with the probability of achieving CR.

**Figure 5:**
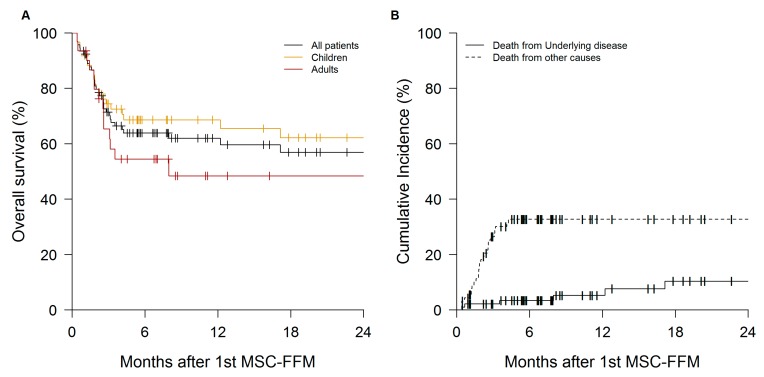
Survival of patients treated with MSC-FFM. (**A**) Six-month overall survival estimates for the entire cohort (solid black line), for children (solid ochre line) and for adults (solid maroon line) were 64% (95% CI 54–74%), 69% (95% CI 58–82) and 54% (95% CI 39–76), respectively. OS was higher than recently published results from comparable cohort that received the best available therapy (Garcia-Cadenas et al. (2017) BMT 52:107–113, Ref. [2]). (**B**) Six-months cumulative incidence of death from underlying disease (solid black line) and from other causes (dashed black line) were 3% (95% CI 0–7) and 33% (95% CI 23–43), respectively.

The six-month overall survival estimate after 6 months was 64% (95% CI 54–75) (Figure 5A) while the cumulative incidence of death from underlying disease was 3.4% (Figure 5B). Data on infections and cause of death, where applicable, are shown in Supplemental Tables S1 and S2.

## 4. Discussion

The data presented here confirm the initial positive impression of acute and long-term R-aGvHD control obtained with MSC-FFM, with seemingly meaningful effects for long-term outcome. Six-month survival estimate of 64% (95% CI 54–75) was observed for the entire cohort and is higher than a recently published OS estimate comparable cohort. In this study, the patients received the best available therapy [2]. While MSC-FFM remained effective in TR-aGvHD, response rates and quality of response were better when it was administered to only SR-aGvHD patients. Whether this is due to a negatively selected patient cohort from which all patients that are responsive to anything at all are missing, or indicates that MSC-FFM works better when aGvHD has been ongoing for a shorter period of time, remains to be seen. However, we interpret the observation as supportive of early use of MSC-FFM as tolerability is very good.

MSC-FFM is in several ways a unique MSC product but which of its unique features is responsible for these promising clinical outcomes is unclear. In earlier work we could demonstrate superior immunomodulatory effects of MSC-FFM compared to individually expanded and then pooled MSCs from the same donors and of the same mitotic age [21]. We therefore posit that the pooling of the BM-MNC prior to MSC generation/extraction by plastic adherence was primarily responsible. Secondly, the high degree of dose-to-dose equipotency ensured that all patients with MSC-sensitive R-aGvHD received active drug, as well as readily interpretable data could be generated. The downside of such a standardized product is that the correlation of product properties and clinical outcomes will not be able to inform about the fingerprint of a “good” MSC or its pharmacological mode of action. 

The clinical observations reported here did not provide hints to the mechanism of action of MSC in R-aGvHD in general or the specific mechanism of action of MSC-FFM in particular. Evidence has been provided that in order to exert the desired pharmacological effects MSCs must undergo a licensing effect in vivo. According to that hypothesis, MSCs respond to the milieu into which they are infused (tissue damage, pro-inflammatory cytokines) by producing soluble factors antagonizing these, e.g., in an inflammatory milieu (such as in aGvHD) [31] they produce anti-inflammatory mediators, exposure to an organ injury signal elicits regeneration-inducing factors, etc. [32]. Thus, pharmacologically speaking, MSCs may be considered a pro-drug. With respect to identification of relevant pharmacological activity and potency assessments on the drug product—of MSCs in general and MSC-FFM in particular—this is not making things easier. After steroid-treatment, lymphocytes are largely depleted, including from aGvHD target organs, so that the assumption that allo-reactivity suppressing activity of MSCs is primarily responsible may fall short. Endothelial damage accompanying aGvHD has been appreciated for many years and is currently emerging as a leading problem of steroid-refractory aGvHD [33,34,35] so that trophic (pro-angiogenic) factors emanating directly or indirectly from MSCs may be equally or even predominantly responsible for observed benefits. Future clinical studies will be needed to advance MSC-FFM to a European Marketing Authorization and should incorporate careful biomarker sub-studies in order to try to address this question. That MSC-FFM represents the pinnacle of what MSCs are capable of is to be doubted. Evidence has been provided that show that MSCs can be substantially enhanced in their functionality by ex-vivo priming, as well as the possibility that, instead of complete cells, extracellular vesicles from MSCs might provide therapeutic usefulness [36].

Certain highly relevant short-comings of the data presented here should be mentioned. We could only collect high-level data and the data were not monitored. Moreover, this is not a clinical trial with defined in- and exclusion criteria and a control cohort, but in- and exclusion was solely directed by the breadth of the label of MSC-FFM as mentioned in Material and Methods. As was shown, partly contradicting several earlier reports, children and adolescents trended towards better responsiveness to MSCs than adults, but this benefit was modest and did not reach statistical significance (Figure 3A). Contributing confounders may be that the sizeable pediatric sub-group differs from the adults in that they were more heavily pre-treated, i.e., likely to represent a higher-risk group. Therefore, it is quite possible that response rates in pediatric and adult patients appear statistically similar in this case series because the children are sicker, as well as the response rates in TR-aGvHD patients are as good as it is because the TR-aGvHD patients are predominantly pediatric patients (who may be more likely to respond to MSCs, according to the literature). This and other issues can only be resolved by further expanding the data base and by performing controlled trials with MSC-FFM. On the other hand, a strength of our data is that it represents real-world experience with MSC for R-aGvHD, in almost exclusively very severe forms of the disease. With 92 recipients of qualitatively identical MSC, ours is also one of the most extensively applied clinical MSC preparations.

## 5. Conclusions

In summary, our data indicated considerable efficacy of MSC-FFM against R-aGvHD. The overwhelming majority of patients treated with MSC-FFM responded with clinical improvement and responses tended to be stable. Moreover, the responding patients had a very satisfactory overall survival. Formal clinical trials, ideally randomized against “standard of care” are needed to confirm this impression.

## 6. Patents

The cell therapy medicine used in this work, MSC-FFM, is protected by patents “Method for MSC generation” EU2975118 (Jan. 2016), US15/326,213 (Aug. 2019), other countries pending.

## Figures and Tables

**Table 1:** Patient characteristics.

	N (Total = 92)	%
**Sex** (f/m)	31/61	34/66
**Diagnosis** (malignant/non-malignant)	69/23	75/25
**Age**		
≤18 [Median (range)], years	61 [7.7 (0.5–18.0)]	66
>18 [Median (range)], years	31 [42.4 (18.4–65.6)]	34
**Donor**		
Matched sibling donor	21	23
Matched unrelated donor	56	61
Mismatched unrelated donor	1	1
Haploidentical	14	15
**Transplant source**		
Bone marrow	41	45
Peripheral blood stem cells	49	53
Umbilical cord blood	2	2
**Conditioning**		
Total body irradiation based	22	24
Treosulfan based	26	28
Busulfan based	21	23
Fludarabine based	21	23
Others	2	2
**Serotherapy**		
None	29	32
Anti-Thymocyte globulin (ATG)	42	46
Campath	17	17
Others	4	4
**GvHD prophylaxis**		
None	10	11
Cyclosporin A (CSA)	12	13
CSA+methotrexate	32	35
CSA+mycophenolate mofetil (MMF)	13	14
Sirolimus+Tacrolimus	5	5
MMF+Tacrolimus	5	5
Others	15	16
**aGvHD severity prior to MSC**		
grade II	3	3
grade III	34	37
grade IV	54	59
Not specified	1	1
**# aGvHD therapies prior to 1st MSC dose**		
1	8	9
2	17	18
3	31	34
4	16	17
5	12	13
6	4	4
≥7	4	4

Patient characteristics are listed; note the over-representation of male patients, of pediatric patients, of patients with underlying malignant disease, severe or very severe aGvHD and heavily pre-treated aGvHD.

## Supplementary Materials

The following are available online at https://www.mdpi.com/2073-4409/8/12/1577/s1, Table S1: Infections, Table S2: Cause of death.
