# Supplementary material for: Children and Adults with Refractory Acute Graft-versus-Host Disease Respond to Treatment with the Mesenchymal Stromal Cell Preparation “MSC-FFM”—Outcome Report of 92 Patients
Source: Cells. 2019 Dec 5;8(12):1577. doi: 10.3390/cells8121577 (PMC6952775; doi:10.3390/cells8121577)
Supplement: Supplementary file 1 [file cells-08-01577-s001.pdf]

Supplemental Tables:

Table S1 - Infections:

Infections	N (%)
**Viral Reactivations in Blood**	
Yes	32 (35)
No	41 (45)
No data	19 (21)
**Viral-related Diseases**	
Yes	19 (21)
No	52 (57)
No data	21 (23)
**Fungal Infections**	
Yes	6 (7)
No	65 (71)
No data	21 (23)
**Bacterial Infections**	
Yes	25 (27)
No	47 (51)
No data	20 (22)
Total	92 (100)

Table S2 – Cause of death

Cause of Death	N (%)
aGvHD related	7 (21)
Infections/Others	20 (59)
Relapse of underlying disease	6 (18)
No data	1 (3)
Total	34 (100)
